# Assessment of urinary 15‐F_2_‐isoprostanes in dogs with urothelial carcinoma of the urinary bladder and other lower urinary tract diseases

**DOI:** 10.1111/jvim.15877

**Published:** 2020-09-16

**Authors:** Andrew D. Woolcock, Adrienne Cheney, Pierre Deshuillers, Deborah Knapp, George E. Moore

**Affiliations:** ^1^ Department of Veterinary Clinical Sciences, College of Veterinary Medicine Purdue University West Lafayette Indiana USA; ^2^ L'École Nationale Vétérinaire d'Alfort Maisons‐Alfort France; ^3^ Department of Veterinary Administration, College of Veterinary Medicine Purdue University West Lafayette Indiana USA

**Keywords:** inflammation, neoplasia, oxidative stress

## Abstract

**Background:**

The 15‐F_2_‐isoprostanes are by‐products of oxidative stress and are increased in the urine of people with lower urinary tract diseases (LUTD), especially urinary neoplasia. Urothelial carcinoma (UC) is the most common urinary neoplasm in dogs. Earlier detection of UC by noninvasive means could lead to improved outcomes. Urinary 15‐F_2_‐isoprostanes potentially could provide this means, but have not been evaluated in dogs with UC.

**Objective:**

The objective of this study was to measure urinary 15‐F_2_‐isoprostanes in dogs with UC and dogs with other LUTD.

**Animals:**

One hundred seventeen dogs: 46 dogs with UC, 30 dogs with LUTD, and 25 control dogs.

**Methods:**

Any dog that was presented with dysuria was eligible for inclusion. Diagnosis of UC was confirmed histologically. Urinalysis was performed in each case, and 15‐F_2_‐isoprostanes quantified by gas chromatography‐negative ion chemical ionization‐mass spectrometry (GC‐NICI‐MS) and normalized to urinary creatinine concentration.

**Results:**

Dogs with urinary diseases (UC + LUTD) had higher median urinary 15‐F_2_‐isoprostanes when compared to control dogs (5.92 ng/mg [range, 0.46‐31.03] vs 3.73 [range, 1.8‐7.98]; *P* = .02). Urinary 15‐F_2_‐isoprostanes were similar in dogs with UC (5.33 ng/mg [range, 0.46‐31.03]) compared to dogs with LUTD (6.29 ng/mg [range, 0.54‐18.93]; *P* = .47) and control dogs (*P* = .06). Dogs with UC had higher qualitative measures of proteinuria (*P* = .004), hematuria (*P* = .01), and epithelial cells on urinalysis (*P* = .002) compared to the other groups.

**Conclusions and Clinical Importance:**

Urinary F_2_‐isoprostanes are not useful for the detection of UC in dogs. Future research could evaluate urinary 15‐F_2_‐isoprostanes as a marker of inflammation in disease progression and prognosis.

**Abbreviations**
GC‐NICI‐MS: gas chromatography‐negative ion chemical ionization‐mass spectrometry
LUTD: lower urinary tract disease
NNLUTD: nonneoplastic lower urinary tract disease
ROS: reactive oxygen species
UC: urothelial carcinoma
UD: urinary disease

## 1 INTRODUCTION

Urothelial carcinoma (UC) is the most common type of urinary bladder cancer in dogs.^1^, ^2^, ^3^, ^4^, ^5^ The prevalence of bladder cancer at university veterinary teaching hospitals has increased steadily over the past 30 years and was reported to be 0.7% in 2010. This increase could be caused by larger numbers of UC cases in the community, enhanced awareness of UC risk in specific breeds leading to more frequent diagnoses, increased referrals to veterinary teaching hospitals, and growing availability of advanced imaging techniques.^1^ In dogs, UC is commonly an invasive tumor, and can affect the urinary bladder, urethra (including the prostatic epithelium in up to 29% of male dogs), and less commonly the ureters and renal pelves.^6^ It is generally a high‐grade tumor and is often locally advanced at the time of diagnosis. This locally advanced state makes the disease challenging to treat. Screening tests that may aid in identifying tumor development would be appealing to allow for earlier detection and intervention. A promising approach has been to evaluate for the presence of the BRAF^V595E^ mutation in urine for the possible detection of UC in dogs.^7^, ^8^, ^9^ Because not all UC in dogs have this mutation, additional tests are being considered, including analysis of copy number variations in segments of chromosomes 13, 19, and 36.^7^, ^8^, ^9^ There is continued interest in exploring inflammatory mediators that could point to the presence of UC.

Some of the risk factors for UC, such as chronic infections, obesity, and chemical exposures could lead to or enhance chronic inflammation of the urinary tract.^1^, ^2^, ^3^, ^4^, ^5^ Chronic inflammation creates a prooxidant state because accumulation of reactive oxygen species (ROS) can injure cellular structural components such as lipids, proteins, and nucleic acids.^10^, ^11^, ^12^ Oxidative stress has been linked to carcinogenesis because of its effects in propagating chronic inflammation and structural cellular damage, eventually causing metaplasia.^10^, ^11^, ^12^, ^13^, ^14^, ^15^ In people, oxidative stress is implicated in the carcinogenesis of tumors of the stomach, colon, liver, and urogenital tract.^11^, ^12^, ^13^ Oxidative stress has been studied in prostatic carcinoma in people, but little is known about the role of oxidative stress in urogenital tumors of dogs.

Isoprostanes are by‐products of oxidative stress, specifically peroxidation of arachidonic acid.^16^ Isoprostanes initially are esterified to phospholipids, and then released as free isoprostanes by phospholipase A_2_.^16^ Although several isoprostane isomers are generated from this interaction, the most extensively studied are the 15‐F_2_‐isoprostanes. The 15‐F_2_‐isoprostanes are highly stable compounds in all tissues, but especially in urine.^16^ By‐products of oxidative damage, such as the isoprostanes, have been utilized to document oxidative stress in a variety of disease states.^16^, ^17^ In people, measurement of urinary isoprostanes is a favored approach for measurement of oxidative stress.^16^

Although cyclooxygenase‐2 (COX‐2) activity and prostaglandin E_2_ (PGE_2_) synthesis have been well documented in dogs with UC, 15‐F_2_‐isoprostanes are not well described in dogs, and studies are limited to dogs with a wide variety of systemic illnesses, but not localized urinary disease.^15^, ^17^, ^18^, ^19^ In people, urinary 15‐F_2_‐isoprostanes are increased in lower urinary tract disease and especially in prostatic carcinoma.^20^, ^21^, ^22^, ^23^ If 15‐F_2_‐isoprostanes are increased in dogs with UC, they would be an appealing screening tool for early detection. Our objective was to measure urinary 15‐F_2_‐isoprostanes in dogs with UC as well as in dogs with nonneoplastic lower urinary tract disease (NNLUTD). We hypothesized that urinary 15‐F_2_‐isoprostanes would be increased in dogs with urinary diseases (UC + LUTD) disease when compared to healthy control dogs. We further hypothesized that urinary 15‐F_2_‐isoprostanes would be increased in dogs with UC when compared to dogs with NNLUTD.

## 2 MATERIALS AND METHODS

### 2.1 Study approval and dogs

The study was approved by the Purdue Animal Care and Use Committee. Dogs presented to the Purdue University Veterinary Teaching Hospital with clinical signs of dysuria were eligible for inclusion in the study. Signs of dysuria included stranguria, pollakiuria, hematuria, and urinary incontinence. Additionally, dogs presented for screening because of breed predisposition for UC were eligible for inclusion. Dogs were excluded from the study if they had received any antiinflammatory medications including nonsteroidal antiinflammatory drugs or glucocorticoids of any form (PO, topical, ophthalmic) within 30 days of presentation. Patients also were excluded if they had received any cytotoxic chemotherapeutic drugs within 30 days of diagnosis. Patients with multiple neoplastic diagnoses were excluded.

Dogs in the study with urinary tract signs were characterized into 2 groups: UC or NNLUTD as described below. In addition to a urinalysis, all dogs were required to have diagnostic imaging either by abdominal ultrasonography or cystoscopy. Abdominal ultrasound images were reviewed by board certified radiologists, and all cystoscopy was performed by diplomates of the American College of Veterinary Internal Medicine. If any mass lesion was noted by either imaging technique, biopsy and histopathology were required to classify the lesion as neoplastic or inflammatory. A diagnosis of UC had to be confirmed histologically; cytologic identification of neoplastic epithelial cells on a urinalysis did not constitute a diagnosis of neoplasia. Dog categorized with NNLUTD had ≥1 of the following: cystoscopic biopsy results of an inflammatory lesion, absence of any masses detected by imaging, and no atypical epithelial cells in the urine in the absence of inflammation.

For the control group, healthy dogs were recruited from the institutional faculty, staff, and students. Health status was confirmed based on collection of patient history, physical examination and urinalysis. Exclusion criteria described above also were applied to this population. Any dog of a high‐risk breed for development of UC (Scottish Terriers, West Highland White Terriers, Wire‐Haired Fox Terriers, Beagles, and Shetland Sheepdogs) was excluded from the control group. Dogs with any history of lower urinary tract disease also were excluded. Follow‐up was performed with the control dogs to assess for any clinical signs of dysuria (described above) that could have developed after the time of sample collection. If dogs developed any clinical signs of dysuria within 3 months of sample collection, they were excluded from the control group.

### 2.2 Sample collection

All dogs enrolled with urinary tract signs had a urine sample collected by 1 of the following methods: sterile urethral catheterization, cystocentesis (if UC was not suspected), or collection during cystoscopy. All control dogs had urine samples collected by midstream voiding because this approach minimized invasiveness in these healthy dogs, and the method was similar to that in dogs with UC. The method of urine collection was determined as appropriate for the case by the attending clinician and study principal investigator. At least 5 mL of urine was collected from each dog. Urine was separated into 2 aliquots. One aliquot was submitted to the institutional clinical pathology laboratory (Clinical Pathology Laboratory, Purdue University Veterinary Teaching Hospital, West Lafayette, IN) for routine urinalysis and microscopic sediment examination. The remaining urine immediately was placed into cryovials and stored at ‐80°C until time of urinary 15‐F_2_‐isoprostane quantification. Urinary 15‐F_2_‐isoprostanes have been shown to be stable for up to 12 months at this temperature.^10^, ^17^

### 2.3 Quantification of urinary 15‐F_2_‐isoprostanes

Gas chromatography‐negative ion chemical ionization‐mass spectrometry (GC‐NICI‐MS) quantification of 15‐F_2_‐isoprostanes was performed at the Eicosanoid Core Laboratory at the Vanderbilt University Medical Center (Nashville, TN) according to their previously published methodology.^16^ Briefly, a stable isotope dilution method was used, in which the F_2_‐isoprostanes were measured against several internal standards for quantification. Isoprostanes were analyzed after conversion to pentafluorobenzyl ester trimethylsilyl ether derivatives. The precision and accuracy of this test are +6% and 96%, respectively.^16^ The lower limit of sensitivity is approximately 20 pg.^16^ Results are reported as ng isoprostane/mg creatinine. Urine creatinine concentration for normalization of the isoprostane concentration was measured by the Jaffe reaction using a commercial chemistry analyzer (Roche COBAS Integra 800, F. Hoffman‐La Roche AG, Basel, Switzerland) by the same laboratory.

### 2.4 Statistical analysis

Sample size was calculated using a comparison of mean urinary F_2_‐isoprostanes in people with and without prostatic carcinoma.^20^ With significance set at *P* < .05 and 80% power, a sample size of at least 20 dogs per group was determined to be necessary to identify a significant difference. The data were analyzed for normality using the Shapiro‐Wilk test. Descriptive statistics are presented as median and range. The Mann‐Whitney *U* test was used to compare concentrations of urinary 15‐F_2_‐isoprostanes and urinalysis results between groups. Spearman‐rank correlation coefficients were calculated for the concentrations of urinary 15‐F_2_‐isoprostanes and the grade severity of UC. Correlation was graded by the following: 0.0‐0.3, no agreement; 0.3‐0.5, poor agreement; 0.5‐0.7, fair agreement; 0.7‐0.9, strong agreement; and 0.9‐1.0, very strong agreement. Values of *P* < .05 were considered significant. Statistics were performed using a commercial software (cMedCalc Software, Mariakerke, Belgium).

## 3 RESULTS

One hundred seventeen dogs were included in the study. Seventy‐six dogs with urinary disease (UD) were included. Of these dogs, 46 were diagnosed with UC (n = 38, high‐grade; n = 3, low‐grade; n = 5, grade not noted), whereas the remaining 30 dogs were diagnosed with NNLUTD. Twenty‐five control dogs were enrolled in the study. No difference was found in age between dogs with UD when considered as a single group compared to healthy control dogs (*P* = .23). Dogs with UC were significantly older than dogs with other LUTD (*P* = .008) and healthy control dogs (*P* = .006). Demographics for these dogs are depicted in Table 1. Of the dogs with NNLUTD, the most common diagnosis was cystitis (n = 16: n = 8, bacterial cystitis; n = 8, lymphocytic and other sterile cystitis; Supporting Information Table S1). Dogs with UC had higher qualitative measures of proteinuria (*P* = .005), microscopic hematuria (*P* = .01), and epithelial cells noted on sediment examination (*P* = .002) compared to the other 2 groups (Table 2).

**TABLE 1:** Demographic information of the dogs with urinary disease, classified based on a diagnosis of urothelial carcinoma (UC) or other nonneoplastic lower urinary tract disease (NNLUTD), as well as demographic information about the group of healthy control dogs

	Age (years)	Sex	Breeds (Listed if ≥2)
All urinary disease (N = 76)	10 [0.33‐18]	52 F (6 intact) 24 M (1 intact)	…
UC (N = 46)	11 [7‐18]^a^	26 F (1 intact) 20 M (0 intact)	Mixed Breed (14), Scottish Terrier (5), West Highland White Terrier (5), Shih Tzu (3), Labrador Retriever (2), Miniature Schnauzer (2), Shetland Sheepdog (2)
NNLUTD (N = 30)	8.5 [0.33‐14]	26 F (5 intact) 4 M (1 intact)	Mixed Breed (8), Boxer (2), Labradoodle (2), Miniature Schnauzer (2)
Control (N = 25)	8 [2–13]	17 F (2 intact) 8 M (1 intact)	Mixed Breed (16), Australian Shepherd (2)

*Notes*: Superscript letters denote a significant difference compared to other values in the same column.

Abbreviations: F, female; M, male.

**TABLE 2:** Urinalysis results from 117 dogs classified as healthy or diagnosed with urothelial carcinoma (UC) or nonneoplastic lower urinary tract disease (NNLUTD)

	Dipstick exam (Results: Negative, 1+, 2+, 3+, 4+)							Sediment exam (Results: None = 0, Few = 1, Many = 2)					
	USG	pH	Prot	Glu	Ket	Bili	Blood	WBC	RBC	Epi Cells	Crystals	Casts	Bacteria
Healthy (N = 25)	1.039 [1.019‐1.065]	6.0 [6.0‐8.0]	0 [0–2]	0 [0–0]	0 [0–0]	0 [0–1]	0 [0‐2]	0 [0‐0]	0 [0‐1]	0 [0‐1]	0 [0‐1]	0 [0‐0]	0 [0–0]
UC (N = 46)	1.010 [1.005‐1.049]	6.5 [6.0‐8.5]	2^a^ [0–4]	0 [0‐1]	0 [0–1]	0 [0‐2]	4^b^ [0‐4]	0.5 [0‐2]	1^c^ [0‐2]	1^d^ [0‐2]	0 [0‐0]	0 [0‐0]	0 [0‐2]
NNLUTD (N = 30)	1.019 [1.005‐1.067]	6.5 [6.0–8.5]	1 [0–4]	0 [0‐0]	0 [0‐0]	0 [0‐3]	1 [0‐4]	0 [0‐2]	0 [0‐2]	1 [0‐1]	0 [0‐0]	0 [0‐0]	0 [0‐2]

*Notes*: Dipstick examination results are presented as either numerical (urine specific gravity, urine pH) or qualitative based on reagent pad (negative through 4+). Sediment examination results are presented as quantitative based on the number seen per high power field, and then classified as none (0), few (1), or many (2). Results are presented as median [range]. Superscripted letters denote a result that is statistically significant when compared to the other groups of dogs in that column.

Abbreviations: Bili, bilirubin; Epi Cells, epithelial cells; Glu, glucose; Ket, ketones; RBC, red blood cell; USG, urine specific gravity; WBC, white blood cell.

**FIGURE 1:**
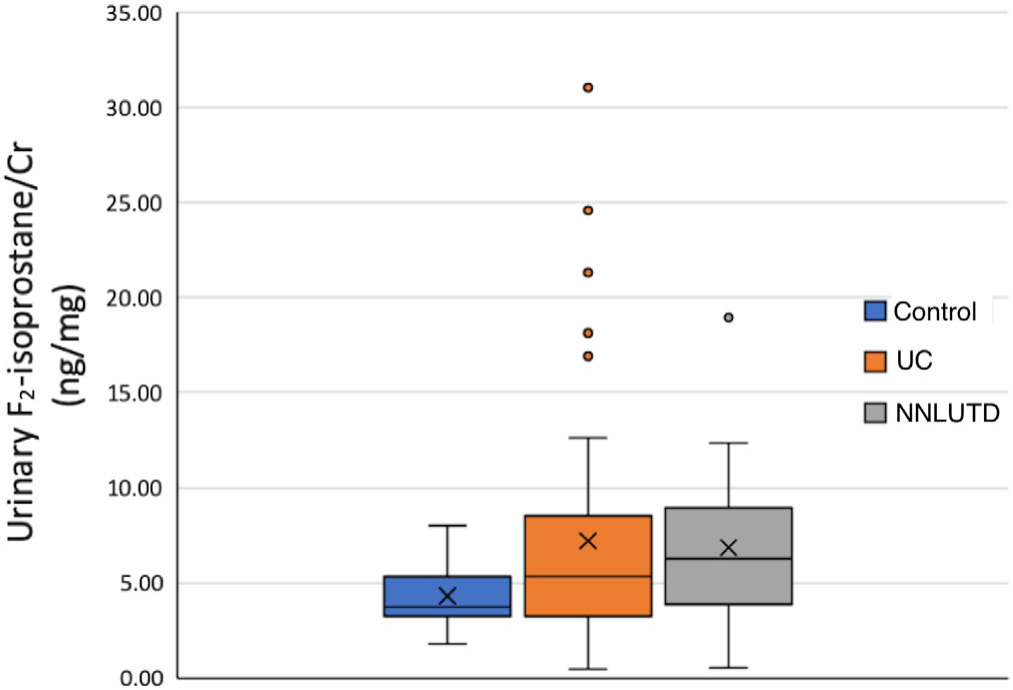
Box and whisker plot depicting the concentration of urinary 15‐F_2_‐isoprostanes in healthy dogs, dogs with UC, and dogs with NNLUTD. The box represents the first and third quartiles with the middle line representing the median. The whiskers represent the range out to 1.5× the IQR, with outliers depicted by individual data points. The median concentration of urinary 15‐F_2_‐isoprostanes was significantly higher in dogs with UC and NNLUTD when compared to healthy dogs (*P* = .03, *P* = .003, respectively). The median concentration of urinary 15‐F_2_‐isoprostanes was not different between dogs with UC compared to dogs with NNLUTD (*P* = .47). IQR, interquartile range; NNLUTD, nonneoplastic lower urinary tract disease; UC, urothelial carcinoma

Concentrations of urinary 15‐F_2_‐isoprostanes were not normally distributed. Median urinary 15‐F_2_‐isoprostane concentrations in dogs with urinary disease (UD) were significantly higher than those of healthy dogs (5.92 ng/mg [range, 0.46‐31.03] vs 3.73 ng/mg [range, 1.80‐7.98], *P* = .02). Median urinary 15‐F_2_‐isoprostane concentrations in dogs with NNLUTD were significantly higher than those of healthy dogs (6.29 ng/mg [range, 0.54‐18.93] vs 3.73 ng/mg [range, 1.80‐7.98], *P* = .01). However, median urinary 15‐F_2_‐isoprostane concentrations in dogs with UC did not significantly differ from those of healthy dogs (5.33 ng/mg [range, 0.46‐31.03] vs 3.73 ng/mg [range, 1.80‐7.98], *P* = .06) or from those of dogs with NNLUTD (*P* = .47, Figure 1). Median urinary 15‐F_2_‐isoprostane concentrations did not correlate with grade of UC (*P* = .86).

## 4 DISCUSSION

Urinary 15‐F_2_‐isoprostane concentrations were increased in dogs with UD, but no difference was found when comparing dogs with UC and those with NNLUTD. The increase in urinary 15‐F_2_‐isoprostane concentration noted in dogs with UD was minor, and several outliers were noted. Substantial overlap was observed in the range of 15‐F_2_‐isoprostane concentrations measured in healthy dogs.

In people, urinary 15‐F_2_‐isoprostane concentrations are increased in a variety of inflammatory diseases. Because of the role of inflammation in bladder cancer, there is interest in measuring isoprostanes for detection of this neoplasm. In people, urinary 15‐F_2_‐isoprostane concentrations have shown promise in diagnosis of prostatic carcinoma, but have not been evaluated in bladder cancer in humans.^20^ Other studies of people with lower urinary tract diseases have used 15‐F_2_‐isoprostanes to calculate relative risk, and this increase in risk has demonstrated the utility of the isoprostanes as a screening tool; relative risk was not evaluated in the study.^21^, ^23^

Results of studies of 15‐F_2_‐isoprostane in humans have been variable, which could be a consequence, in part, of different methods used for urinary 15‐F_2_‐isoprostane measurement. The isoprostanes represent a family of compounds derived from the metabolism of arachidonic acid, of which there are multiple isomers.^16^ The 15‐F_2_‐isoprostanes are the most readily measured, but other isomers that were not assessed in our study may be produced by inflammatory and neoplastic diseases. Urinary 15‐F_2_‐isoprostanes can be measured by multiple methods including radioimmunoassay, enzyme‐linked immunosorbent assay (ELISA), and mass spectroscopy (by gas or liquid chromatography).^16^, ^24^ In people, a study using radioimmunoassay did not identify a difference in urinary 15‐F_2_‐isoprostane concentrations between patients with prostatic carcinoma and those with other lower urinary tract disease, whereas other studies identified a difference when mass spectroscopy was used.^20^, ^21^ The GC‐NICI‐MS and ELISA methods were found to have poor agreement when comparing urinary 15‐F_2_‐isoprostane concentrations in people, dogs, and cats.^25^, ^26^, ^27^ Currently, gas chromatography/mass spectroscopy is the recommended method for quantification of urinary 15‐F_2_‐isoprostanes in people and domestic species, which is the method used in our study.^25^, ^26^, ^27^ It is acknowledged that, in our study, the method of urine collection in the dogs with lower urinary tract signs varied from case to case. It was considered unsafe to collect urine by cystocentesis if mass lesions were present or UC was suspected. Although the effects of urine collection method have not been studied in dogs, in other species the method of urine collection has not altered the results of the 15‐F_2_‐isoprostane assay.^25^, ^27^

Inflammation of the lower urinary tract has been studied in people and dogs, and biomarkers of inflammation including cyclooxygenase (COX‐1 and COX‐2) expression, PGE_2_ concentrations, and cytokine profiles have been used to measure the impact of diseases such as urinary tract infections and urinary neoplasia.^14^, ^15^, ^29^ In people, activity of the arachidonic acid pathway is increased in the presence of urinary tract neoplasia.^14^, ^15^, ^29^ In dogs, COX expression was noted in 5/9 cases of prostatic carcinoma, and PGE_2_ concentrations were increased in 21/22 dogs with UC.^18^, ^19^ Our study only measured urinary 15‐F_2_‐isoprostanes, presuming they would reflect the inflammatory and prooxidant environment created by inflammation or cancer. The minimal change in urinary 15‐F_2_‐isoprostanes in UD of dogs does not lessen the likelihood that inflammation damages the urothelium, but rather reflects that the isoprostanes are too narrow in diagnostic scope. A recent study of UC in dogs identified differences in the lipid profiles of neoplastic urothelial cells when compared to healthy urothelium.^30^ This finding may indicate that a more direct measurement of lipid profiles could offer better insight into the tumor microenvironment than a by‐product of lipid peroxidation such as the 15‐F_2_‐isoprostanes.

In people, the 15‐F_2_‐isoprostanes were found to be useful as a pretreatment prognostic factor for prostatic carcinoma.^20^, ^21^, ^22^, ^23^, ^29^ Most prostatic carcinomas in men however are adenocarcinomas, and most carcinomas in dogs are urothelial cancers arising from the ducts. With their frequency in dogs, UC (bladder, urethra, or prostate) was the target neoplasm in our study. Although these tumor types share urothelium and proximity, it is possible the prooxidant factors that would contribute to lipid peroxidation in UC of the prostate could differ from those of UC of the bladder. Therefore, future studies could include larger numbers of dogs with UC of the prostate to evaluate isoprostane concentrations in this more specific tumor subset. Oxidative stress in tumors, in general, often is attributed to the influence of inflammatory cytokines.^10^, ^11^, ^12^ However, in prostatic carcinoma of men it is theorized that the effect of androgens also contributes to reactive oxygen species (ROS) production and a prooxidant environment.^15^ If true, the largely neutered and primarily female population of dogs included in our study would not experience these androgenic effects, and therefore the downstream effects of oxidative stress would be expected to differ. Future studies could include larger numbers of dogs and a more balanced population of male and female dogs to allow better assessment of sex differences and possible prostatic involvement.

Our study had some limitations. The use of urinary 15‐F_2_‐isoprostanes as a sole biomarker of oxidative stress did not allow for a complete evaluation of the prooxidant and antioxidant factors that could be imbalanced in the presence of disease. This limitation is difficult to address because no consensus currently exists as to how best to evaluate oxidative stress in domestic species, but often >1 measurement is utilized.

In conclusion, urinary 15‐F_2_‐isoprostanes do not appear to be a meaningful biomarker for evaluating dogs presenting with clinical signs localized to the lower urinary tract. Further research will be required to more thoroughly evaluate the presence of oxidative stress in dogs with lower urinary tract disease and urinary tract neoplasia.

## CONFLICT OF INTEREST DECLARATION

George Moore serves as Consulting Editor for Experimental Design and Statistics for the Journal of Veterinary Internal Medicine. He was not involved in review of this manuscript.

## OFF‐LABEL ANTIMICROBIAL DECLARATION

Authors declare no off‐label use of antimicrobials.

## INSTITUTIONAL ANIMAL CARE AND USE COMMITTEE (IACUC) OR OTHER APPROVAL DECLARATION

Authors declare no IACUC or other approval was needed.

## HUMAN ETHICS APPROVAL DECLARATION

Authors declare human ethics approval was not needed for this study.

## Supporting information


**Table S1** Dogs diagnosed with NNLUTD, the corresponding diagnosis/diagnoses, and the diagnostic tests utilized to obtain diagnosis. AUS, abdominal ultrasound; HP, histopathology; NNLUTD, nonneoplastic lower urinary tract disease.
