# Supplementary material for: Assessment of urinary 15‐F2‐isoprostanes in dogs with urothelial carcinoma of the urinary bladder and other lower urinary tract diseases
Source: J Vet Intern Med. 2020 Sep 16;34(6):2454–9. doi: 10.1111/jvim.15877 (PMC7694844; doi:10.1111/jvim.15877)
Supplement: Supplementary file 1 — Table S1 Dogs diagnosed with NNLUTD, the corresponding diagnosis/diagnoses, and the diagnostic tests utilized to obtain diagnosis. AUS, abdominal ultrasound; HP, histopathology; NNLUTD, nonneoplastic lower urinary tract disease. [file JVIM-34-2454-s001.pdf]

Supplemental Table. Dogs diagnosed with NNLUTD, the corresponding diagnosis/diagnoses, and the diagnostic tests utilized to obtain diagnosis. AUS, abdominal ultrasound; HP, histopathology; NNLUTD, non-neoplastic lower urinary tract disease.

Dog	Diagnosis/Diagnoses	Method(s) of Diagnosis
1	Urinary bladder mass (Non-diagnostic)	Cystoscopy; HP
2	Urinary incontinence (open for cause); Bacterial cystitis	Cystoscopy; Urine culture
3	Urinary bladder proprial fibrosis	Cystoscopy; HP
4	Chronic cystitis	Cystoscopy; HP
5	Thickened/proliferative urethra (Non-diagnostic)	Cystoscopy; HP
6	Necrohemorrhagic cystitis	Cystoscopy; HP
7	Proliferative cystitis; Calcium oxalate urocystoliths	Cystoscopy; HP; Stone analysis
8	Bacterial cystitis (chronic)	Cystoscopy; HP; Urine culture
9	Recurrent bacterial cystitis	Cystoscopy; Urine culture
10	Anogenital cleft	Cystoscopy
11	Cystitis and lymphoid follicle	Cystoscopy; HP
12	Recurrent bacterial cystitis	Cystoscopy; Urine culture
13	Recurrent bacterial cystitis; Recurrent urocystoliths (struvite)	AUS; Stone analysis; Urine culture
14	Persistent vaginal septum; Recurrent bacterial cystitis	Cystoscopy; Urine culture
15	Urocystoliths (calcium oxalate)	Cystoscopy, Stone analysis
16	Urocystoliths (calcium oxalate)	Cystoscopy, Stone analysis
17	Ectopic ureter, bacterial cystitis	Cystoscopy; Urine culture
18	Reflex dysynergia	Cystoscopy
19	Inflammatory urothelial polyp, non-suppurative vaginitis	Cystoscopy; HP
20	Ectopic ureters	Cystoscopy
21	Lymphoplasmacytic cystitis and urethritis	Cystoscopy; HP
22	Urinary incontinence (open for cause)	AUS; Urinalysis
23	Ectopic ureter	Cystoscopy
24	Urinary bladder polyp, chronic cystitis	Cystoscopy; HP
25	Ulcerative cystitis	Cystoscopy; HP
26	Calcium oxalate urocystoliths	Cystoscopy, Stone analysis
27	Primary renal hematuria	Cystoscopy; Urinalysis
28	Lymphocytic follicular cystitis	Cystoscopy; HP
29	Polypoid and hemorrhagic cystitis; Cystitis glandularis	Cystoscopy; HP
30	Right intramural ectopic ureter	Cystoscopy
